# Straw Lumber—A New Industry

**Published:** 1881-06

**Authors:** 


### STRAW LUMBER—A NEW INDUSTRY.

There can be no question that straw lumber is admirably adapted to many kinds of finishing work, barrels, tables and counter tops, fine doors and ornamental work, and we are assured that it can be produced and sold in competition with the finer grades of pine, or in competition with wide walnut, at about one-half the price of the latter. The standard manufacture is in widths of 32 inches, a length of 12 feet, and a thickness corresponding to that of surfaced boards. These dimensions may be varied to suit such orders as may be given, and embrace any width, length or thickness. Unlike lumber, however, narrower widths are the most costly. The straw lumber may be ripped with the hand-saw or upon the buzz-saw; may be run through the sticker for the manufacture of moldings, and takes a nail or screw about as well as oak. It may be finished with varnish or with paint, and is susceptible of a high polish. It is water and practically fire-proof, being manufactured under five hundred degrees of heat, and we are assured has been boiled for some hours without any apparent change of structure. Its tensile strength is greater than walnut or oak, and its weight about one-fifth greater than the former when dry. It is made from any kind of straw, including hemp and flax fibre—in fact, from any material that will make pulp—and a ton of straw will produce 1,000 feet of boards. The pulp is rolled into thin sheets, a number of which, corresponding with the thickness of the lumber desired, are placed together with a peculiar cement which is claimed to be water-proof, and are then rolled under a pressure sufficient to amalgamate them into a solid mass, which may be worked with the plane if desired.

We look for valuable results in the future in the manufacture of lumber from what is practically a waste material, but which will be produced in endless quantities so long as the United States maintains its character as a grain-producing country.—*North-western Lumberman.*

---

"I THOUGHT you took an interest in my welfare," said William. "No, sir," replied Susan; "only in your farewell."

SCENE, a butcher's stand. Butcher: "Come, John, be lively; now break the bones in Mr. Williams' chops, and put Mr. Smith's ribs in the basket for him." John (briskly): "All right, sir; just as soon as I've sawed off Mrs. Murphy's leg."

